# Body Composition Evaluation Issue among Young Elite Football Players: DXA Assessment

**DOI:** 10.3390/sports5010017

**Published:** 2017-02-23

**Authors:** César Leão, Mário Simões, Bruno Silva, Filipe Manuel Clemente, Pedro Bezerra, Miguel Camões

**Affiliations:** 1 Escola Superior de Desporto e Lazer, Instituto Politécnico de Viana do Castelo, 4960-320 Viana do Castelo, Portugal; msimoes@esdl.ipvc.pt (M.S.); silvabruno@esdl.ipvc.pt (B.S.); filipeclemente@esdl.ipvc.pt (F.M.C.); pbezerra@esdl.ipvc.pt (P.B.); joaocamoes@esdl.ipvc.pt (M.C.); 2 Instituto Politécnico da MAIA-Grupo de Investigação para o Desporto, Educação e Saúde (GIDES), 4475-690 Maia, Portugal; 3 Instituto de Telecomunicações, Delegação da Covilhã, 6201-001 Covilhã, Portugal; 4 Research Center in Sports Sciences, Health and Human Development (CIDESD), 5000-801 Vila Real, Portugal

**Keywords:** body fat evaluation, DXA, BIA, young, football

## Abstract

Accurate assessment of body composition is an important issue among athletes. Different methodologies generate controversial results, leading to a deep uncertainty on individual exercise prescriptions. Thus, this study aims to identify the differences between field methods, such as bioelectrical impedance (BIA) and skinfold assessment, with a clinical method, highly accurate, dual-energy X-ray absorptiometry (DXA), among elite young football players. Thirty-eight male football players with a mean (sd) age of 16.7 (0.87) years, involved in the Portuguese national competition of U16 (*n* = 13) and U19 (*n* = 25), were evaluated and objective measures of body composition, muscle strength and football skills were collected by trained specialists. Body composition was assessed using BIA (Tanita BC-418, Tanita Corp., Tokyo, Japan), in agreement with all the evaluation premises. Additionally, all athletes were evaluated using the clinical method DXA (Hologic Inc., Waltham, MA, USA). Among the U19 athletes, three skinfold sites (SKF) were assessed: chest, abdomin and thigh. The Spearman correlation coefficients and the mean difference between methods were calculated. The agreement between both methods was analyzed using Bland-Altman plots. Among the evaluated athletes, lower mean values of body fat % were found using BIA as a method of body composition assessment compared with DXA (12.05 vs. 15.58 for U16; 11.97 vs. 14.16 for U19). Despite the moderate correlation between methods (*r* = 0.33) to estimate the percentage of total fat, the median of the difference (DXA vs. BIA) was relevant in clinical terms, with 2.90% and 1.47% for U16 and U19 athletes, respectively. Stronger correlations were found between the sum of the SKF and DXA fat estimation (*r* = 0.68). The Bland-Altman plots showed a clear underestimation in the evaluations using the BIA, namely among athletes with better body composition profiles (8%–12% of fat). Using BIA, an underestimation of body fat assessment was observed among 94.5% of the athletes with less than 12% body fat mass. Among the evaluated athletes, fat mass was underestimated at a median value of 2.21% using BIA in comparison with DXA. The sum of the SKF showed a stronger correlation with the reference method (DXA) (*r* = 0.68) than BIA.

## 1. Introduction

There is a significant relationship between competitive success in several sports contexts and certain anthropometric characteristics [1]. In football, we find a heterogeneity in anthropometric and physiological characteristics that makes it impossible to isolate single pre-requisites [2]. However, some physiological characteristics such as aerobic [3] and anaerobic capacity, strength, power [4] and speed [5] are closely related to body composition among elite football athletes [6,7,8]. 

The body composition in athletes is a conditioning factor influencing their performance, particularly in jumping ability [9] and in the capacity to execute specific tasks rapidly [10], independently of gender, age and ethnicity. Even small changes in body fat % may have a major impact on the ability to perform anaerobic movements [11]. The assessment of body composition can provide valuable information about the changes observed in athletes during the season [12]. In addition, body composition data may be important in the selection procedures of young athletes, allowing a comparison with reference values, and from there building an athlete development program [13]. Moreover, an incorrect assessment of the body composition may lead to difficulties in prescribing a proper eating plan because of the pressure to achieve a target body fat value [14]. 

The body composition assessment provides information of particular relevance, with the percentage of body fat being the most valued parameter [14], to either athletes and/or coaches [3,9,15] to determine the optimal body composition. Acknowledging the impact that the manipulation of body composition has on athletic performance, ideally it should take place as soon as possible in the sports season, before the competitive period [16]. Therefore, it is a common practice to assess body composition early in the season and later on in response to training and dietary interventions [8,17], expecting a change in body weight and especially in fat mass [18]. Obtaining a type-specific body composition is directly associated with individual performance, and it is currently recognized as a significant challenge to individualize and periodize the athlete’s development process [16].

Despite the importance given to body composition, it remains difficult to obtain an accurate analysis of the percentage of body fat. The available tools for body composition assessment are either inaccurate or supported in data of weak validity, the opposite of what we assume most of the time [19]. The importance of assessing body fat in athletes notwithstanding, there is still no method that offers 100% accuracy [20]. The choice of method should consider several factors, including technical issues, such as security, validity, evaluation of precision and reliability. Additionally, there are other factors to consider, in particular practical factors such as availability, financial implications, portability, invasion of privacy, time availability and technical expertise to conduct the method [17,21]. All available techniques have some inherent advantages and disadvantages, either in methodology, interpretation of data or the assumptions that are made from the same. Hence, the adherence to the prerequisites for each of the techniques is a key requirement [20]. Even methods considered as a reference may have limitations when you change the behaviors before assessment that can impact hydration status [22]. The use of different methods in the evaluation of body composition provides inconsistent results, very often leading to difficulties and doubts in the individual training plan prescription [23].

Currently, the most accepted method for evaluating healthy adults is dual-energy X-ray absorptiometry (DXA) [21], but it is considered costly or inaccessible for most teams, especially young teams [24]. On the other hand, bioelectrical impedance (BIA) has become increasingly popular as an analysis tool of body composition due to its ease of use, portability and low cost [17].

This study describes the differences between field methods, such as BIA and skinfold assessment, and a clinical method, the highly accurate dual-energy X-ray absorptiometry (DXA), among elite young football players.

## 2. Materials and Methods

### 2.1. Participants

An observational study was conducted with 38 male football players with mean (sd) age of 16.7 (0.87) years, involved in the Portuguese national competition of under-16 (U16) (*n* = 13) and under-19 (U19) (*n* = 25). Study participants were invited to visit the Escola Superior de Desporto e Lazer-Instituto Politécnico de Viana do Castelo to be evaluated on several sports performance determinants. The participants were asked to maintain habitual daily food and water intake during the period of study.

At the time of the evaluations, athletes were on a maintenance phase of the National U16 and U19 championship. These football athletes train a mean of 6 h/week having an average of 6 years of football experience with systematized training. Table 1 describes the characteristics of the athletes, stratified by competitive age (U16 and U19), regarding age, height, weight, body mass index, BIA percentage of fat and DXA percentage of fat.

The research was approved by the technical-scientific council of the Instituto Politécnico de Viana do Castelo and all intervenient signed the Free and Clarified Consent Form according to the Declaration of Helsinki [25].

### 2.2. Anthropometrics

One week before of the laboratory assessments, it was required to the technical staff of the team that some characteristics on the athletes needed to be preserved in order to reduce the error in the estimation of the different body compartments [26]. All participants were dress light clothing and stood barefoot, with eyes directed straight ahead. Athletes’ height was measured to the nearest 0.1 cm with a portable stadiometer (SECA 217, Hamburg, Germany). 

### 2.3. Body Composition

The body composition was analysed with multi-frequency BIA (Tanita^®^ BC-418, Tanita Corp., Tokyo, Japan). This test provides a complete analysis of weight, body mass index, body fat and fat mass percentage, fat free mass and total body water. Before the assessment, the trained specialists manually inserted data on body type profile (athlete format), age, and measured height into the system. The subjects wiped their feet and stood on the weighing platform without bending their knees [26]. All the participants were in agreement with all the evaluation premises, in order to reduce the error in the estimation of the different body compartments: like fasting or stay 4 h without food or drink, absence of exercise in the prior day, the absence of alcohol or diuretic drinks, the need of a stable temperature of 23 °C in the room [26].

In addition, among all the athletes, body composition was evaluated using the clinical method DXA through a General Electric Hologic Discovery scanner (Hologic Inc., Waltham, MA, USA), as stated by the manufacturer specification and with a certified and experienced DXA operator. DXA provides information on three compartments of body composition, according to the terminology: percentage of (%) fat mass, lean mass or the fat-free soft tissue and bone mineral content. Athletes assumed a stationary, supine position on the scanning bed with both arms pronated by their side. The DXA operator manually assisted the young players in order to: (1) straighten the head; (2) position of the shoulders, pelvis and legs; (3) place both arms in pronation by their side; and (4) fix feet together using strapping [27]. Only the data from whole body % of fat mass and subtotal (without head) % of fat mass was considered for the analyses.

### 2.4. Skinfolds

In a subsample, among the U19 athletes (*n* = 25), three sites skinfolds (SKF) were collected, two times (to the nearest 0.1 mm), with a Harpenden caliper (British Indicators, Ltd., London, UK), following the recommendations of the International Society for the Advancement of Kineanthropometry [28]: chest, abdominal and thigh sites. The mean value of the two evaluations was calculated, and the sum of the three SKF was considered.

### 2.5. Statistical Analysis

A descriptive analysis was performed regarding the anthropometric characteristics, namely fat % among different methods used: BIA, DXA. Non parametric tests were used and the Wilcoxon test was applied to verify the differences in continuous variables between competitive level (U16 and U19). The median values were found to analyse the differences between the reference method—DXA and both field methods, BIA and SKF assessment.

Spearman’s correlation coefficients were calculated to describe the relationship between methods. The agreement was illustrated by plotting the differences between the methods against their mean using the Bland-Altman’s graphics [29]. 

All data sets were tested for each statistical technique and corresponding assumptions and performed using SPSS software (IBM Corp. Released 2014. IBM SPSS Statistics for Windows, Version 23.0, Armonk, NY, USA).

## 3. Results

The recruited 38 male football athletes had an overall mean (sd) age of 16.8 (0.87) years. The older athletes were heavier (kg) than the younger ones (69.81 vs. 66.25, *p* = 0.056). Supported by the body composition reference method, these athletes were significantly different regarding their body composition. We can see that despite the higher value of absolute weight in U19, there was a significant DXA lower body fat % (14.16 vs. 15.58, *p* = 0.041). Regardless of this, the BIA method did not show significant differences in body fat % among the competitive levels (11.97 vs. 12.05, *p* = 0.913)*,* as shown in Table 1.

A moderate correlation (Table 2) was found between the percentage of fat found with BIA and the percentage of fat measured with DXA (*r* = 0.335, *p* = 0.040). Considering the sum of the three skinfolds (SKF) valued in the U19 athletes, we observed a stronger correlation between SKF scores and the percentage of fat measured with DXA (*r* = 0.683, *p* < 0.001).

Despite the moderate significant correlations found between the field methods and DXA, we observed that the mean difference between the methods was clinically relevant, as shown in Table 3. Fat mass was underestimated by a median value of 2.21% using BIA in comparison with DXA. 

**Figure 1:**
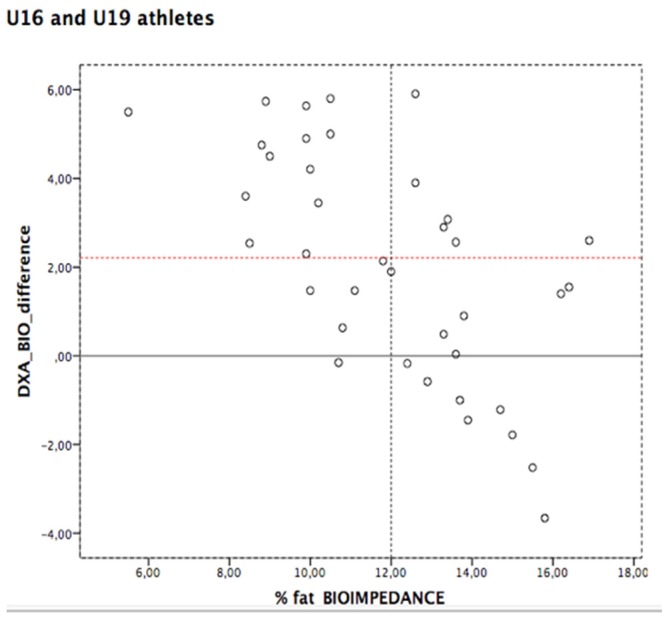
Bland-Altman plots (red line represents the median value of the difference between methods). U16—Under 16; U19—Under 19; DXA—dual-energy X-ray Absorptiometry; BIA—bioelectrical impedance; %—percentage.

Bland-Altman plots (Figure 1) showed a clear tendency regarding the evaluations with BIA. We can see that the smaller the value of fat % measured with BIA, the bigger the difference with the DXA assessment.

## 4. Discussion

Among elite youth football athletes, we observed moderate correlations between field methods and DXA on body composition assessment. The use of BIA in clinical practice has been validated for various populations [30], but the comparison with a reference method such as DXA, in athletes, has few published studies to date.

BIA is a safe and non-invasive method based on the difference of the electrical conductivity of body fat and fat-free mass [17]. Despite BIA being widely used to estimate body composition, there is still some difficulty in accurately assessing the percentage of body fat from this method [20]. One of the difficulties lies in the need to comply with some assumptions that interfere with the final estimates, such as fasting or spending 4 h without food or drink, the absence of exercise the previous day, the absence of alcohol or diuretic drinks, and the need for a stable temperature of 23 °C in the room [26]. These requirements may interfere with the hydration status, and hence interfere in the correct body composition assessment [20]. Even small changes, such as the fasting period before assessment, can lead to changes in the fat mass estimates by BIA [31]. Another important aspect is that manufacturers do not supply the reference population or the equations in the device used in our study, which makes it difficult to compare with other studies.

In the literature, moderate correlations between BIA and DXA were found [32]. However, these results do not necessarily mean there is a good agreement between methods. In that regard, the present study found a high median value of the difference between the methods (DXA vs. BIA), resulting in fat mass underestimation (2.21%) when using the field method BIA. Other studies, albeit conducted in non-athletes, comparing BIA and DXA reported a systematic underestimation of the body fat percentage by BIA, especially in lean subjects, which is consistent with our results [33,34,35]. In addition, in non-athletes as well, with different body profiles, we found an overestimation of the body fat percentage, especially in overweight subjects [36,37,38,39].

The weight increase, especially in fat-free mass, may be a desired goal, but a body fat increase as large as 2% may lead to decreased performance, for example in vertical jumping [40]. For this reason, evaluation with BIA can lead to misguided training and diet plans in the pursuit of a lower body fat percentage [13,14]. 

The Bland-Altman plots showed a distinct tendency in the evaluations using BIA, namely among athletes with a better body composition profile (8%–12% of body fat). A clear underestimation of body fat assessment using BIA was observed among 94.5% of the athletes with less than 12% body fat. These results show some agreement with the existing evidence in young athletes. Krzykała (2016) and Sillanpää (2013) have shown that BIA overestimates athletes’ body fat percentage, especially in those with lower percentages of body fat in DXA scans. Additionally, BIA underestimated the fat percentage in athletes with more body fat as assessed by DXA [41,42]. For this reason, the use of BIA can lead to deviations from the reference method which may be a limitation to its use in individual evaluation [20].

The use of SKF to evaluate body composition is accepted as valid for athletes [43,44]. It is possible to compare the values we found in U19 athletes with what would be expected in football players [1]. In addition, the use of SKF has been shown to be an alternative that correlates much better with DXA than BIA in athletes [45,46]. Further, although it was not the main goal of this study, we also found that there was a higher correlation between SKF and DXA in these young football players. This can lead to a further discussion about the existing methods to evaluate body composition and their uses in the field.

Despite the small sample size, this observational study provides objective data collected by trained specialists, and the correlations between body composition assessment methods were supported in the DXA comparison, increasing the reliability of the results. Nevertheless, one of the limitations on body composition evaluation and comparison between studies is that there are several brands and types of devices to assess the body fat percentage and fat-free mass [47]. For this reason, in practical terms, it becomes difficult to make comparisons between studies because different devices are used. In addition, the validity of the prediction equations is dependent on how similar the population of interest is to the reference population in which the prediction equations were tested. This assumption could affect the results supported by BIA measurements and could have an impact on the differences found between tested methods. However, having taken into account the different determinants of imprecision that affect the final body composition estimations, this study made the athletes’ objective measurements in the same day with trained physicians and under standardized protocols.

## 5. Conclusions

The main findings of our study suggest that despite being a valid method for use in athletes, there must be caution in the way the results obtained with BIA are interpreted, even taking into account the method’s moderate correlation with DXA. Since there is already a validation of the use of skinfolds to estimate body composition in athletes, which is also a very accessible method that is easy to implement, with fewer determinants of imprecision on the final estimates and with a high correlation with DXA [48], it is our suggestion to provide SKF data collection to assess and control body composition among elite athletes. 

## Figures and Tables

**Table 1:** Sample characteristics.

	U16 (*n* = 13)		U19 (*n* = 25)		*p*-Value
	Mean	sd	Mean	sd	
Age (years)	15.77	0.44	17.28	0.54	<0.001
Height (cm)	174.62	5.68	175.16	6.40	0.927
Weight (kg)	66.25	5.03	69.81	5.39	0.056
BMI (kg/m^2^)	21.65	1.17	22.76	1.52	0.025
BIA fat mass (%)	12.05	2.66	11.97	2.66	0.903
DXA fat mass (%)	15.58	2.03	14.16	1.91	0.041
Sum SKF (mm)	-	-	36.12	8.19	-

Notes: Significance level *p* < 0.05; U16—Under 16; U19—Under 19; BMI—Body Mass Index; kg—kilograms; kg/m^2^—kilograms per square meter; %—percentage; mm—millimeters; DXA—dual-energy X-ray Absorptiometry; BIA—bioelectrical impedance; SKF—skinfold.

**Table 2:** Spearman correlation coefficients between methods.

	DXA Fat Mass (%)	*p*-Value
BIA fat mass (%)	0.335	0.040 *
Sum of the three skinfolds (mm)	0.683	<0.001 **

Notes: * Significant correlation at the 0.05 level (2-tailed); ** Significant correlation at the 0.05 level (2-tailed); DXA—dual-energy X-ray Absorptiometry; BIA—bioelectrical impedance; %—percentage; mm—millimeters.

**Table 3:** Descriptive analysis (mean, standard deviation, and median values) on % of body fat estimation among methods and the differences between them.

	Mean	sd	Median
BIA fat (%)	12.0	2.62	12.20
DXA fat (%)	14.06	2.20	13.64
DXA fat-BIA fat (%)	2.06	2.55	2.21

DXA—dual-energy X-ray Absorptiometry; BIA—bioelectrical impedance; %—percentage
